# Development and mechanical testing of a short intramedullary nail for fixation of femoral rotational osteotomy in cerebral palsy patients

**DOI:** 10.1186/1475-925X-10-57

**Published:** 2011-06-28

**Authors:** Rodrigo G Pagnano, Rodrigo Okubo, Jose B Volpon

**Affiliations:** 1 Laboratory of Bioengineering, School of Medicine of Ribeirão Preto, University of São Paulo, Brazil; 2 Department of Orthopaedics, School of Medicine of Ribeirão Preto, University of São Paulo, Brazil

## Abstract

**Background:**

Rotational osteotomy is frequently indicated to correct excessive femoral anteversion in cerebral palsy patients. Angled blade plate is the standard fixation device used when performed in the proximal femur, but extensile exposure is required for plate accommodation. The authors developed a short locked intramedullary nail to be applied percutaneously in the fixation of femoral rotational osteotomies in children with cerebral palsy and evaluated its mechanical properties.

**Methods:**

The study was divided into three stages. In the first part, a prototype was designed and made based on radiographic measurements of the femoral medullary canal of ten-year-old patients. In the second, synthetic femoral models based on rapid-prototyping of 3D reconstructed images of patients with cerebral palsy were obtained and were employed to adjust the nail prototype to the morphological changes observed in this disease. In the third, rotational osteotomies were simulated using synthetic femoral models stabilized by the nail and by the AO-ASIF fixed-angle blade plate. Mechanical testing was done comparing both devices in bending-compression and torsion.

**Results:**

The authors observed proper adaptation of the nail to normal and morphologically altered femoral models, and during the simulated osteotomies. Stiffness in bending-compression was significantly higher in the group fixed by the plate (388.97 ± 57.25 N/mm) than in that fixed by the nail (268.26 ± 38.51 N/mm) as torsional relative stiffness was significantly higher in the group fixed by the plate (1.07 ± 0.36 Nm/°) than by the nail (0.35 ± 0.13 Nm/°).

**Conclusions:**

Although the device presented adequate design and dimension to fit into the pediatric femur, mechanical tests indicated that the nail was less stable than the blade plate in bending-compression and torsion. This may be a beneficial property, and it can be attributed to the more flexible fixation found in intramedullary devices.

## Background

Femoral anteversion is the anterior projection of the femoral neck related to the coronal plane. It develops prenatally and is approximately 40° at birth. Then, gradually, it decreases during the postnatal growth to a position of approximately 15° at skeletal maturity [1]. Failure of anteversion to decrease is a common feature in children with cerebral palsy and is caused by muscle imbalance. This static deformity, associated with overactivity of internal rotators of the hip, results in an internally rotated gait that can lead to functional and cosmetic problems [2]. Excessive femoral anteversion and medial rotation deformity of the hip are best corrected with femoral derotation osteotomy (FDO). Angled blade plate is the standard fixation device used when FDO is performed in the proximal femur. Although it provides rigid fixation that allows early postoperative mobilization, extensile exposure is required for plate accommodation and it may be oversized in relation to the hypoplastic bone. As FDO usually is done as part of multilevel surgery in cerebral palsy, it would be desirable to reduce its morbidity through a minimally invasive technique.

Intramedullary rigid nail has widely been employed for fixation of femoral fractures and osteotomies in adults. There is concern about using these implants in skeletally immature patients due to the risk of avascular necrosis and growth disturbances of the proximal femur [3-6]. Studies that used a lateral trochanteric entry point for intramedullary nailing in children and adolescents demonstrated low complication rate, without cases of necrosis or evidence of growth changes [7,8]. The nails used in these studies are long, extending throughout all the extension of the femoral shaft with a variable length between 220 and 420 mm and, when indicated for rotation osteotomy fixation, adopt a mid-diaphyseal level of bone section. The purpose of this study is the development of a short femoral nail for the fixation of rotation osteotomy to be performed at a subtrochanteric level through a percutaneous technique and to test its mechanical properties.

## Methods

### Prototype development

**Figure 1:**
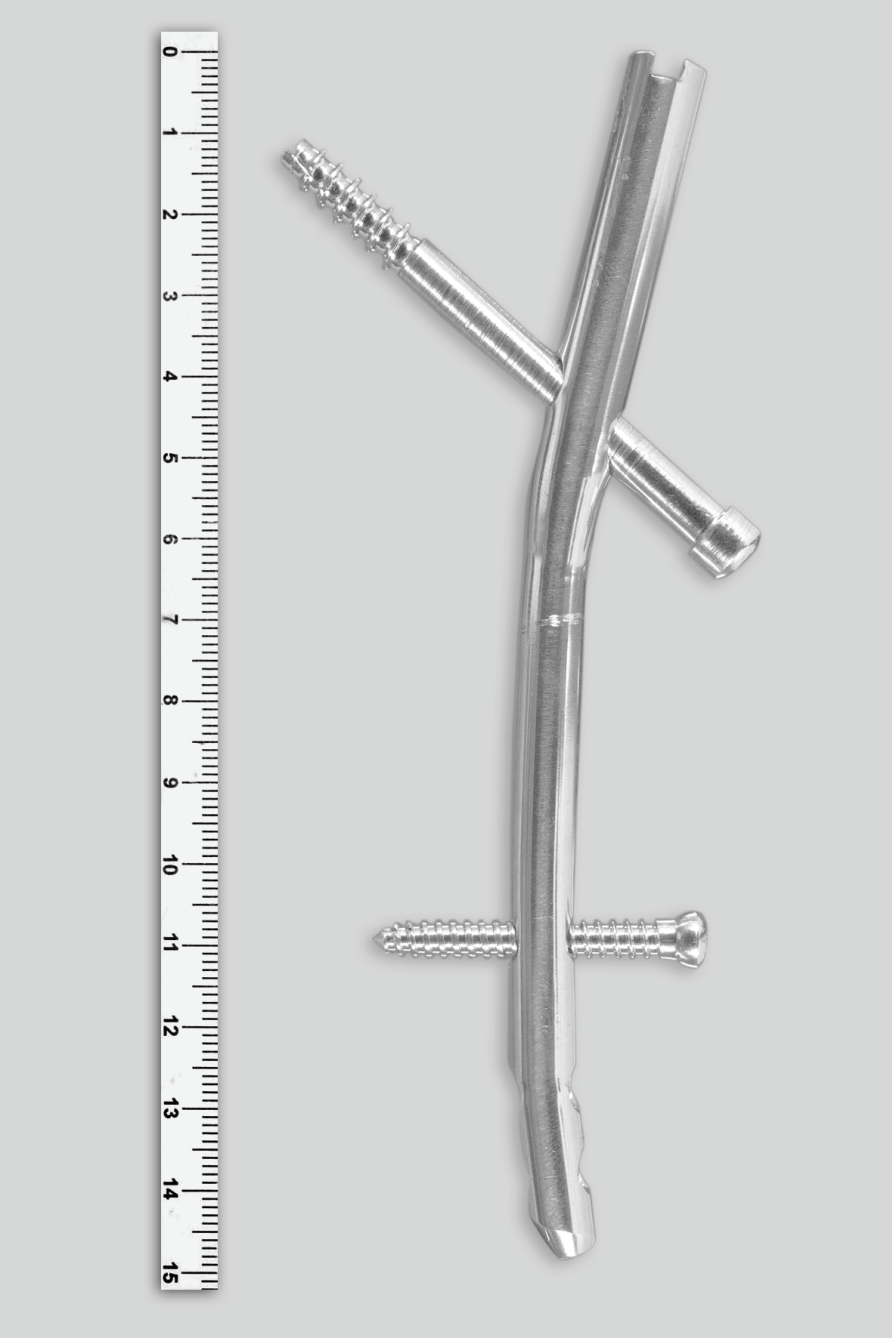
**Nail prototype**. Nail prototype with the 6.5-mm cannulated proximal locking screw and the 4.5-mm cortical distal locking screw.

In the first stage, a prototype was designed based on the shape and the dimensions of femurs of ten-year-old patients. After approval by the hospital's Institutional Review Board, the authors reviewed plain radiographs taken in 25 male patients followed up with unilateral Perthes' disease. These radiographs included the pelvis and both proximal femurs, and the unaffected side was used for the measurement of femoral medullary canal diameter at five levels. These measurements were adjusted with a 15% reduction corresponding to the magnification of the radiographs (Table 1). The nail prototype was designed to be inserted at a lateral trochanteric entry point and have two locking screws, one proximal and the other distal to the osteotomy level. A prototype was made in stainless steel (Figure 1), and it consisted of a cannulated nail with a proximal segment with 10.0 mm of external diameter and a distal segment with 8.0 mm of external diameter. The nail had a 11° apex medial proximal bend, 50.0 mm from the proximal end and a 10° apex medial distal bend, 40.0 mm from the distal end. In the lateral plane, the nail was straight for inserting it in either the right or the left femur. The proximal segment had a 6.5-mm locking hole directed obliquely from distal to proximal and from lateral to medial, resulting in a 135° cephalic inclination of the proximal screw to the longitudinal axis of the nail. The proximal locking screw was a 6.5-mm-diameter cannulated screw with a partial thread for cancellous bone fixation. The distal segment of the prototype had three 4.5-mm locking holes perpendicular to the long axis of the implant, and the most proximal was used as distal locking hole. The distal locking screw was a 4.5-mm-diameter solid screw with thread for cortical bone fixation. The nail prototype measured 147.0 mm in length, based on the average distance measured on the radiographs between the lateral region of the greater trochanter and the site of narrowing of the medullary canal. A complete set of instruments was made for the preparation of the femoral medullary canal and to insert and lock the nail.

**Table 1:** Femoral medullary canal diameter

*Patient*	*Region 1*	*Region 2*	*Region 3*	*Region 4*	*Region 5*
1	12.17	9.57	9.57	8.70	9.57
2	11.74	10.43	10.43	9.57	10.00
3	15.22	13.91	13.04	11.74	11.74
4	13.04	11.30	10.87	10.00	10.00
5	13.04	11.30	10.43	10.00	10.43
6	13.04	10.43	8.70	8.26	8.26
7	12.17	10.43	10.00	9.57	9.57
8	15.22	13.91	12.61	12.17	11.74
9	12.61	11.74	11.30	9.57	10.00
10	14.78	12.61	11.74	11.30	10.87
11	16.09	12.61	11.74	10.00	10.00
12	15.65	14.35	12.61	12.61	12.61
13	11.74	10.43	9.57	8.26	8.26
14	16.52	14.35	12.61	10.87	11.74
15	13.04	10.87	10.43	9.57	9.57
16	11.30	9.57	8.26	8.26	9.57
17	13.04	10.43	9.57	8.70	8.26
18	10.43	9.57	8.26	8.26	8.26
19	13.04	10.87	10.43	10.00	9.57
20	10.43	8.70	8.26	8.26	9.57
21	13.91	13.04	11.30	9.57	9.57
22	12.17	10.43	9.57	9.57	10.00
23	12.17	11.30	10.87	10.00	10.43
24	12.17	10.87	10.43	9.57	9.57
25	12.61	10.43	10.00	9.13	9.13
					
mean	13.09	11.34	10.50	9.74	9.93
					
median	13.04	10.87	10.43	9.57	9.57
					
standard deviation	1.65	1.59	1.39	1.22	1.14
					
minimum	10.43	8.70	8.26	8.26	8.26
					
maximum	16.52	14.35	13.04	12.61	12.61

Region 1: 3 cm distal to the lesser trochanter; Region 2: 5 cm distal to the lesser trochanter; Region 3: 6 cm distal to the lesser trochanter; Region 4: 8 cm distal to the lesser trochanter; Region 5: 10 cm distal to the lesser trochanter

Diameter (mm) of the medullary canal at five levels related to the lesser trochanter measured on radiographs of the uninvolved femur of patients with Legg-Calvé-Perthes disease at the age of 10 years.

### Rapid prototyping of synthetic femoral models

For final adjustments of the nail prototype to morphological changes found in cerebral palsy patients, the authors get synthetic femoral models based on reconstructed tomographic images. After approval by the hospital's Institutional Review Board, the authors reviewed preoperative computed tomography (CT) scans of ten-year-old patients with cerebral palsy that were ordered as part of surgical planning for other purposes.

**Figure 2:**
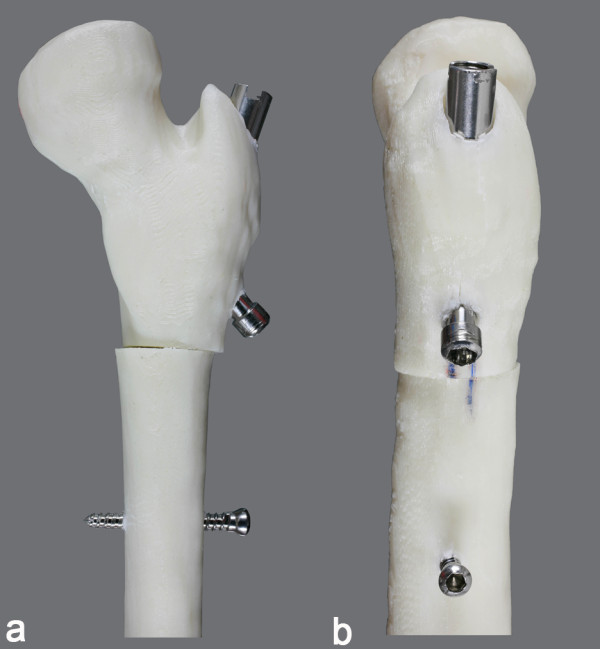
**Simulated rotational osteotomy performed in a morphologically altered femoral model**. Simulated rotational osteotomy fixed by the nail prototype, performed in a morphologically altered synthetic femoral model based on reconstructed tomographic image of a 10-year-old patient with cerebral palsy. Frontal view (a) and lateral view (b).

CT image data (Dicom files) were processed using Mimics program (Materialise NV, Belgium) where the region of interest was isolated and translated into a 3D wireframe model of the proximal femur. Then, physical models were built in thermoplastic material (ABS*plus*™) by a 3D printer (Dimension Elite, Stratasys, Eden Prairie, MN, USA). The nail prototype was inserted in these models, and osteotomies were simulated to assess the need for adjustment in its final design (Figure 2).

### Femoral rotational osteotomy simulation

**Figure 3:**
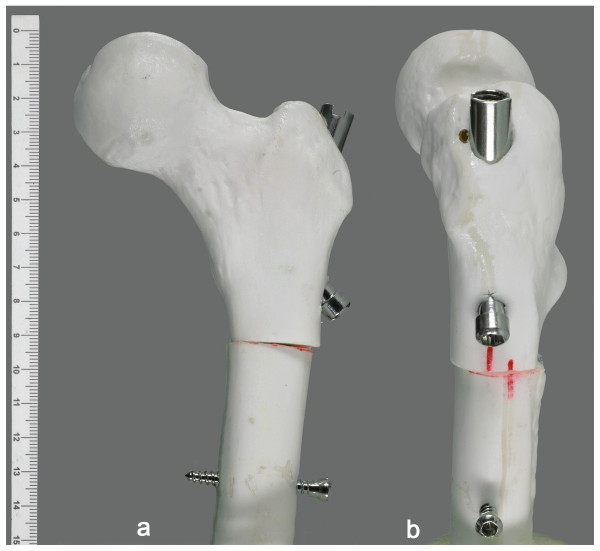
**Simulated rotational osteotomy performed in a morphologically normal femoral model and fixed by the nail prototype**. Simulated rotational osteotomy fixed by the nail prototype, performed in a synthetic adolescent-sized femoral model. Frontal view (a) and lateral view (b).

The nail prototype was inserted and a subtrochanteric rotational osteotomy was performed in synthetic adolescent-sized epoxy femoral models (Pacific Research Laboratories Inc., Vashon WA, USA) (Figure 3). The femoral models measured 35 cm in length, with a central canal diameter of 9.5 mm and head diameter of 45 mm.

### Nail implantation

An opening was made with an awl in the lateral aspect of the grater trochanter and a 2.0-mm guide wire was passed into the medullary canal. Over this guide wire, a 9.0-mm flexible cannulated reamer was driven into the medullary canal until it reached the mid-diaphyseal portion of the model, followed by a 11.0-mm reamer for the proximal portion. After removing remains from the medullary canal, the nail was inserted manually with an insertion handle. After connecting the proximal external locking guide to the insertion handle, a guide pin was inserted toward the center of the femoral neck and through the proximal nail hole, followed by a cannulated drill bit and a cannulated screw, completing the proximal locking. A transverse osteotomy was then created at the subtrochanteric level with use of a 2.0-mm drill bit to make several holes and was completed with an osteotome. After completing the osteotomy, the distal segment was externally rotated to decrease the anteversion angle of the femoral model in 20°, guided by external markings made on the cortex. The distal segment was then locked in this position with a 4.5-mm cortical screw transfixing both model cortices and the distal locking hole.

### Mechanical testing

**Figure 4:**
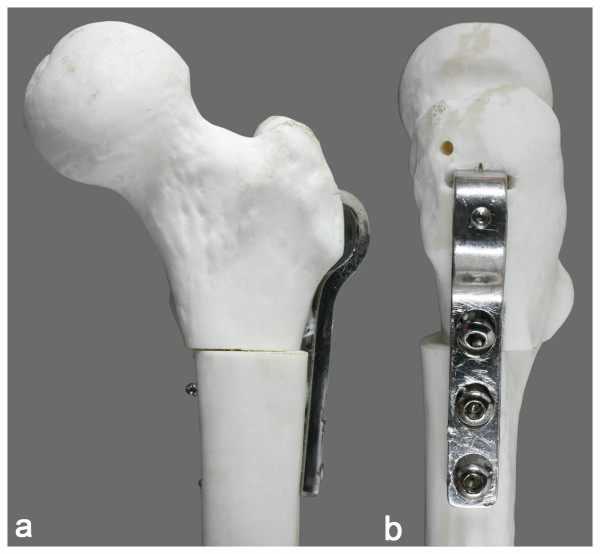
**Simulated rotational osteotomy performed in a morphologically normal femoral model and fixed by a 90° angled-blade plate**. Simulated rotational osteotomy fixed by a 90° angled-blade children's plate, performed in a synthetic adolescent-sized femoral model. Frontal view (a) and lateral view (b).

Ten adolescent-sized femoral models (Pacific Research Laboratories Inc., Vashon, WA, USA) were used for mechanical testing. In five of them, a subtrochanteric osteotomy was performed and fixed by the nail prototype as described earlier. In other five models, the authors performed osteotomies at the same level, fixed by a 90° angled-blade children's plate (Synthes, Switzerland) with the usual technique (Figure 4). Each fixed model was cut in the mid-diaphyseal region, and both groups were submitted to nondestructive bending-compression and torsion tests.

### Bending-compression test

**Figure 5:**
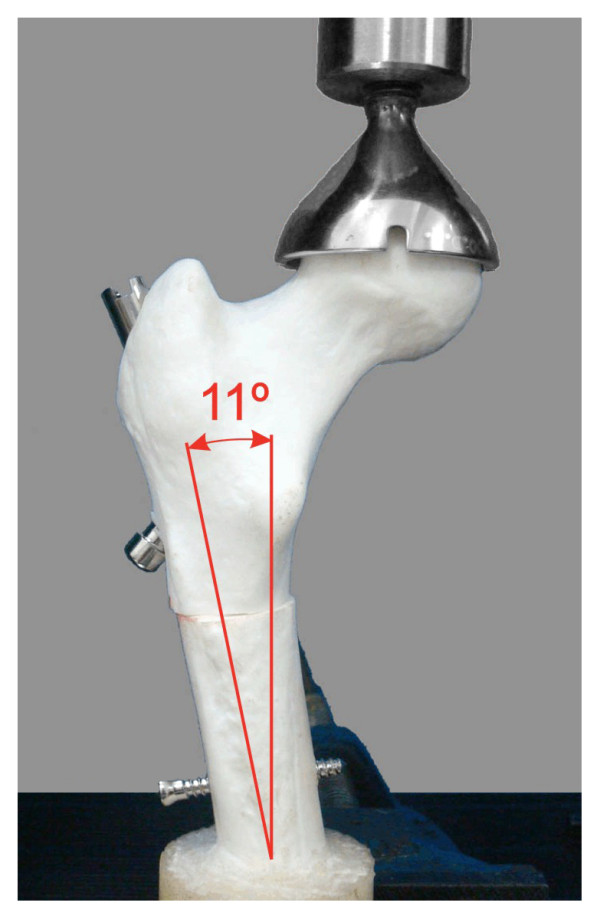
**Femoral model within the testing machine for bending-compression test**. Photograph demonstrating a femoral model within the testing machine for bending-compression test at 11° in adduction.

For the bending-compression test, the distal extremity of the femoral models was embedded in acrylic cement at 11° in adduction, and the models were loaded proximally by means of a socket-shaped accessory (Figure 5) connected to a 200-kgf load cell on a mechanical testing machine (EMIC-100 KN, Brazil). The load was applied vertically at the rate of 0.1 mm/sec, until 1000 N or a 10.0-mm linear displacement was reached. The models were tested three times each, removing the whole setup between the runs.

### Torsion test

**Figure 6:**
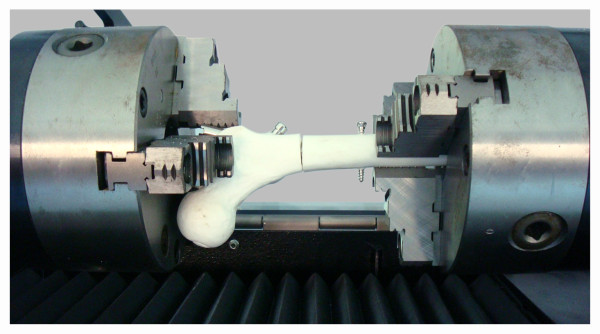
**Femoral model within the torsion testing machine**. Photograph demonstrating a femoral model placed horizontally in the testing machine for torsion test.

For torsion tests, the femoral models were placed horizontally in a torsion testing machine (Instrom 55MT, Instrom, Norwood, MA, USA.), with the main axis visually aligned to the torsional axis of the machine (Figure 6). The distal segment of the model was clamped by the fixed part of the testing machine, while the proximal segment was clamped by the rotating part in the intertrochanteric region. The load was applied at a speed of 10°/min until a maximum torque of 2 Nm or 20 degrees of angular deformation was reached. The models were tested three times each, removing the whole setup between the runs.

### Statistical analysis

A comparison was made among the two fixation groups using a linear regression model with mixed effects (fixed and random effects), used for data analysis when answers for each specimen are grouped and is assumed that independent observations in the same group are not adequate [9]. For using this model, it is necessary that the residue has normal distribution with zero mean and constant variance. The model adjustment was done using PROC MIXED of SAS 9.1 software (SAS Institute, Gary, NC, USA).

Graphics were made using the software R version 2.8.0 (The R Foundation for Statistical Computing).

The significance level P < 0.05 was adopted.

## Results

The shape and the dimensions of the nail prototype were adequate for insertion in synthetic adolescent-sized femoral models. Limitations to nail progression into the medullary canal were not observed during implantation, as well as macroscopic fractures in the model cortex. After the osteotomy was completed, the distal segment could be freely rotated with the nail inside the canal. A slight varus deviation of the proximal segment was observed after osteotomy fixation. The same findings were observed when the nail was implanted in prototyped femoral models with morphological changes due to cerebral palsy. The set of insertion instruments was adequate and the external guides showed good correspondence with the nail locking holes, either for proximal or distal locking.

Gross inspection showed different fixation properties between the devices. Models fixed by the nail showed slight macroscopic rotational and telescoping movements during manipulation, whereas this was not observed in models fixed by the blade plate.

**Figure 7:**
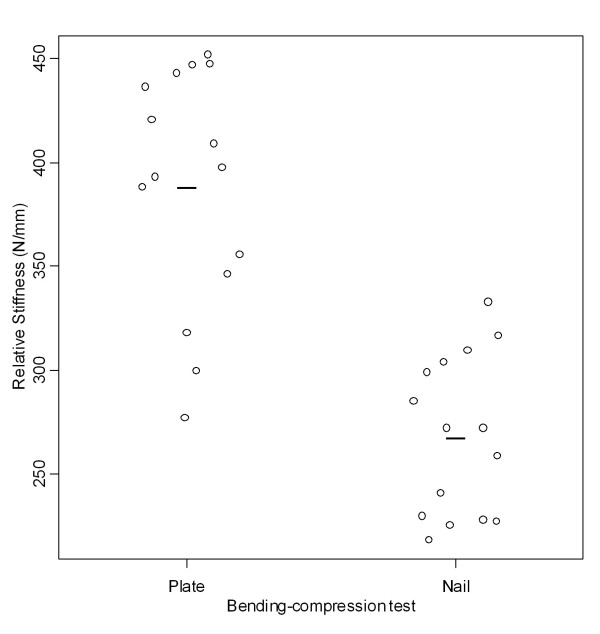
**Relative stiffness found in the bending-compression tests**. Graphic representation of relative stiffness values found in the bending-compression tests according to the groups fixed with plate and nail.

**Figure 8:**
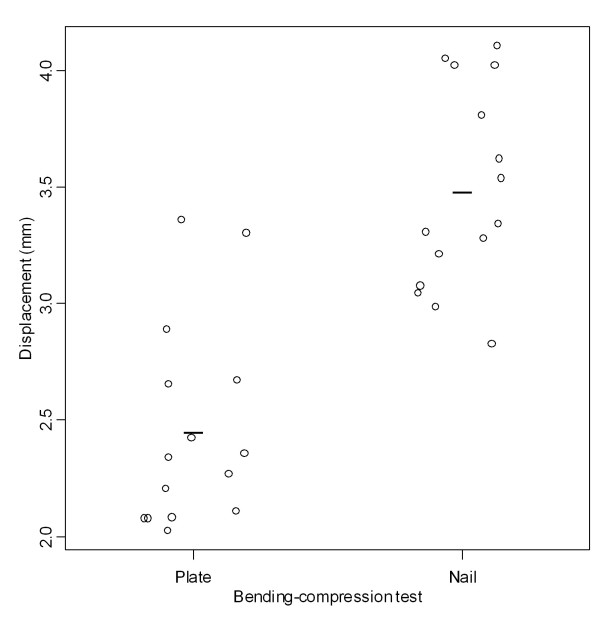
**Linear displacement found in the bending-compression tests**. Graphic representation of linear displacement values found in the bending-compression tests according to the groups fixed with plate and nail.

Relative stiffness in bending-compression was significantly higher (p < 0.001) in the group fixed by the plate (388.97 ± 57.25 N/mm) than that fixed by the nail (268.26 ± 38.51 N/mm) (Tables 2 and 3, Figure 7). Displacement in bending-compression for a 1000-N load was significantly smaller (p < 0.001) in the group fixed by the plate (2.46 ±0.44) than that fixed by the nail (3.48 ± 0.43 mm) (Tables 2 and 3, Figure 8).

**Table 2:** Bending-compression tests

Measure	Implant	Mean (SD)	Mean difference	CI 95%		
				LL	UL	*p-value*
Relative stiffness (N/mm)	Nail	268.26 (38.51)	-120.71	-154.84	-865.76	<0.001
	Plate	388.97 (57.25)				
Linear displacement (mm)	Nail	3.48 (0.43)	1.03	0.71	1.34	<0.001
	Plate	2.46 (0.44)				

Mean (standard deviation), mean difference, and confidence interval of the mechanical parameters used in bending-compression tests for comparison among the two fixation groups.

**Table 3:** Values of the bending-compression tests

Bending-compression test						
**Implant**	**Specimen**	**Repetition**	**Relative stiffness (N/mm)**	**Mean (±SD)**	**Linear displacement (mm)**	**Mean (±SD)**

		1	225.47		4.11	
					
	1	2	228.10	227.88 (2.30)	4.05	4.06 (0.04)
					
		3	230.06		4.02	
	
		1	272.62		3.35	
					
	2	2	272.38	276.87 (7.56)	3.22	3.20 (0.15)
					
		3	285.60		3.05	
	
		1	316.71		3.08	
					
Nail	3	2	304.34	306.73 (9.03)	3.28	3.22 (0.13)
					
		3	299.13		3.31	
	
		1	258.77		3.54	
					
	4	2	309.96	300.71 (38.17)	2.99	3.12 (0.37)
					
		3	333.41		2.83	
	
		1	218.60		4.02	
					
	5	2	227.81	229.13 (11.25)	3.81	3.82 (0.20)
					
		3	240.99		3.62	

		1	420.74		2.21	
					
	1	2	447.78	435.13 (13.60)	2.08	2.13 (0.07)
					
		3	436.87		2.11	
	
		1	299.74		3.36	
					
Plate	2	2	318.07	298.44 (20.32)	2.89	3.19 (0.26)
					
		3	277.50		3.30	
	
		1	393.32		2.36	
					
	3	2	355.89	365.29 (24.71)	2.65	2.56 (0.18)
					
		3	346.67		2.67	
	
		1	388.25		2.43	
					
	4	2	409.20	413.62 (27.84)	2.34	2.28 (0.18)
					
		3	443.41		2.09	
	
		1	447.12		2.08	
					
	5	2	451.98	432.38 (29.83)	2.03	2.12 (0.13)
		3	398.05		2.27	

Values found in the bending-compression tests according to the groups fixed with plate and nail and variability between repetitions of the mechanical tests.

**Figure 9:**
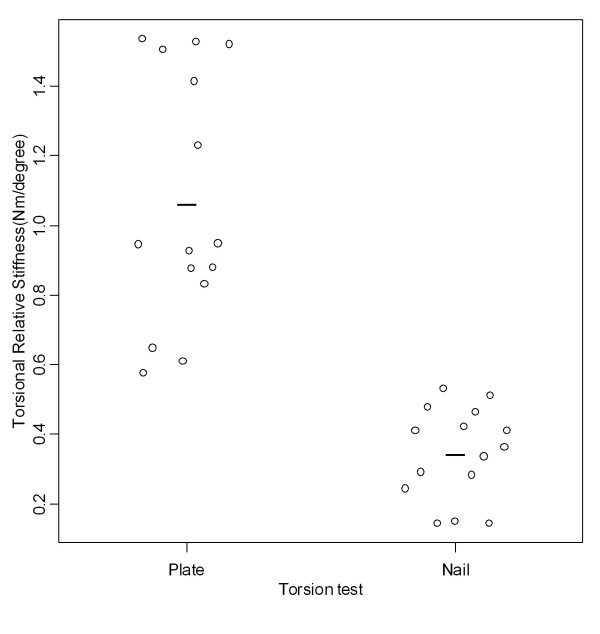
**Relative torsional stiffness found in torsion tests**. Graphic representation of relative torsional stiffness values found in torsion tests according to the groups fixed with plate and nail.

**Figure 10:**
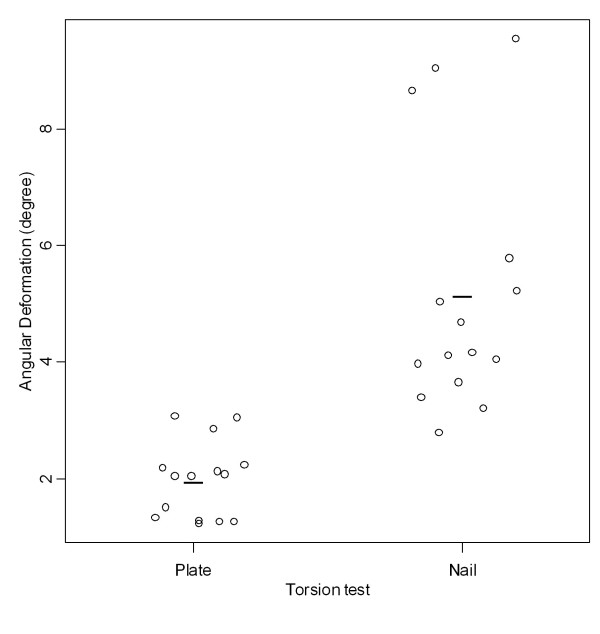
**Angular displacement found in the torsion tests**. Graphic representation of angular displacement values found in the torsion tests according to the groups fixed with plate and nail.

Relative torsional stiffness was significantly higher (p < 000.1) in the group fixed by the plate (1.07 ± 0.36 Nm/°) than that fixed by the nail (0.35 ± 0.13 Nm/°) (Tables 4 and 5, Figure 9). Angular displacement for a 2-Nm torque was significantly smaller in the group fixed by the plate (1.98 ± 0.65°) than in that fixed by the nail (5.16 ± 2.18°) (Tables 4 and 5, Figure 10).

**Table 4:** Torsion tests

Measure	Implant	Mean (SD)	Mean difference	CI 95%		
				LL	UL	*p-value*
Torsional relative stiffness (Nm/degree)	Nail	0.35 (0.13)	-0.72	-0.88	-0.55	<0.001
	Plate	1.07 (0.36)				
Angular dislocation (degree)	Nail	5.16 (2.18)	3.18	2.11	4.26	<0.001
	Plate	1.98 (0.65)				

Mean (standard deviation), mean difference, and confidence interval of the mechanical parameters used in torsion tests for comparison among the two fixation groups.

**Table 5:** Values of the torsion tests

Torsion test						
**Implant**	**Specimen**	**Repetition**	**Relative torsional stiffness (Nm/degree)**	**Mean (±SD)**	**Angle (degree)**	**Mean (±SD)**

		1	0.37		3.66	
					
	1	2	0.41	0.37 (0.04)	3.21	3.64 (0.42)
					
		3	0.34		4.05	
	
		1	0.42		4.13	
					
	2	2	0.51	0.41 (0.11)	2.80	4.00 (1.13)
					
		3	0.29		5.05	
	
		1	0.41		5.22	
					
Nail	3	2	0.48	0.48 (0.06)	3.40	4.26 (0.91)
					
		3	0.54		4.18	
	
		1	0.25		5.79	
					
	4	2	0.29	0.33 (0.12)	4.69	4.82 (0.91)
					
		3	0.47		3.99	
	
		1	0.15		8.67	
					
	5	2	0.15	0.15 (0.00)	9.05	9.09 (0.44)
					
		3	0.15		9.54	

		1	0.61		3.07	
					
Plate	1	2	0.58	0.61 (0.04)	3.06	3.00 (0.11)
					
		3	0.65		2.87	
	
		1	0.93		2.08	
					
	2	2	0.95	0.94 (0.01)	2.05	2.06 (0.02)
					
		3	0.94		2.05	
	
		1	1.23		1.52	
					
	3	2	1.53	1.43 (0.17)	1.23	1.34 (0.15)
					
		3	1.53		1.27	
	
		1	0.88		2.14	
					
	4	2	0.88	0.86 (0.03)	2.19	2.19 (0.05)
					
		3	0.83		2.24	
	
		1	1.41		1.35	
					
	5	2	1.51	1.48 (0.06)	1.29	1.30 (0.04)
					
		3	1.52		1.27	

Values found in the torsion tests according to the groups fixed with plate and nail and variability between repetitions of the mechanical tests.

## Discussion

Orthopedic surgical management of cerebral palsy has been modified in recent years as a single-event multilevel approach was introduced. Addressing hip, knee, ankle, and foot contractures and deformities in a single surgical session tends to improve outcome by reducing rehabilitation periods and need for repeated procedures [10-12]. This approach requires, besides an experienced team, that each procedure have its morbidity decreased. Success with the multiple procedures that include bone surgery depends, to a large extent, on obtaining rigid fixation of osteotomized segments to allow for early healing, comfort, and rapid remobilization in the postoperative period [13]. Rotation osteotomy, to correct excessive femoral anteversion, is frequently indicated as part of multilevel surgery in cerebral palsy patients.

There are controversies regarding the ideal site for femoral rotation osteotomy, whether proximal or distal. Some authors demonstrate comparable safety and efficacy for proximal and distal levels [14,15], while others consider that proximal osteotomy allows a more accurate correction [16,17]. When performed in the proximal femur, the standard fixation device is the angled-blade plate, developed by AO/ASIF (Association for Study of Internal Fixation). Its implantation requires extensile exposure with periosteal stripping and concomitant blood loss and could result in more morbidity [18]. Another aspect is that sometimes it may be overdimensioned, mainly in patients with neuromuscular disorders, where size, shape, and bone quality could be altered [19].

This review studied the development and mechanical testing of an alternative method for stabilizing this proximal osteotomy, a short intramedullary nail to be performed with percutaneous technique.

Restrictions on the use of rigid intramedullary nails in skeletally immature patients are due to the risk of causing necrosis of the femoral head and growth disorders in the proximal femur [3-6]. Using a lateral trochanteric entry point for the nail, as described in previous studies [7,8,20], avoids such complications. According to a study [21], after the age of eight years, at least 50% of the growth of the greater trochanter occurs by an appositional mechanism. Gordon et al. [7] hypothesize that the crucial injury to proximal femur during intramedullary nailing through the tip of the trochanter is damage to the medial aspect of the trochanteric physis. The presence of proximal and distal bends in the present study's prototype allowed more lateral access on the greater trochanter and easier insertion of the nail. However, adopting this entry point may have resulted in varus of the proximal portion of the instrumented models, as described previously [22,23], as the insertion point is not collinear with the axis of the femoral medullary canal. The authors believe that this slight varus may not be a problem, whereas patients with CP usually have excessive valgus of the femoral neck.

The prototype differs from the available nails, used for fracture and osteotomy fixation in children and adolescents [7,8,18,20,24], for being short and advocating a subtrochanteric level of osteotomy. As this level is adopted, a long nail is not needed. In theory, a long nail can be more difficult to transverse the entire medullary canal if it has an irregular diameter and bendings are present, as may occur in patients with neurological sequelae. Using a short nail carries an additional advantage as the distal locking system becomes more accurate and an image intensifier may not be necessary. Distal locking in long nails is accomplished using a free-hand technique under fluoroscopic guidance and is time consuming. The use of the image intensifier, however, is not completely avoided in this technique due to the need for precise location of the proximal locking screw in the center of the femoral neck and for keeping a safe distance between the screw and the growth plate of the femoral head.

The authors tried to avoid using nails of different diameters for simplifying the technique and decreasing costs. A new implant should be simple to produce and to place. The nail developed has small diameter to be employed in the largest possible number of cases, besides functioning as an axis to allow rotation of the osteotomized fragments with the implant within the medullary canal.

The number of distal locking screws has also been discussed. The initial designs included the presence of two screws near the end of the nail. However, studies on fixation properties of distal screws in adult femoral nails showed that a single screw located more proximally could be sufficient and also decreases stress concentrations at the nail end [25]. Predicting implant removal after bone union, the use of a single screw could represent an additional advantage by eliminating another point of weakness in the bone. Therefore, the authors chose a single distal locking screw located more proximally.

Synthetic femoral models obtained from cerebral palsy patients by rapid prototyping were used to evaluate the nail adaptation to altered femurs and the need for adjustments. The findings of the visual analysis of these models were consistent with the morphological changes expected in cerebral palsy patients. Simulations of rotational osteotomies performed in these models have demonstrated the suitability of the nail design for implantation within the medullary canal and to the rotation and the fixation of the fragments. Similar findings were observed when the authors used adolescent-sized bone models of normal characteristics.

Mechanical fixation properties of the nail were compared with the standard fixation device, the AO-ASIF fixed-angle plate. For this, it was necessary to simulate the behavior of these devices in similar situations. A synthetic bone model was chosen due to difficulty in obtaining samples of pediatric cadaveric bones. These models have been used in biomechanical studies because of the consistency between the samples and their minimal variability in size and physical properties [26-28].

Gross observations and manual manipulation of the instrumented femoral models revealed slight rotation and telescoping movements between the segments in the nail fixation group. This was not observed in the plate fixation group. These findings were expected as the type of fixation provided by the nail does not produce interfragmentary compression.

The parameters set for bending-compression testing were based on estimates of forces acting on the frontal plane during single leg stance [29]. Relative stiffness up to the application of a 1000-N load and the linear dislocation occurring in this situation were the parameters adopted. Despite knowledge of the biomechanics of the hip in the frontal plane, little is known about the action of forces in other planes [30], hence the difficulty in establishing the torsion parameters. The authors adopt a maximum torque of 2 Nm in torsion tests, as was used in a biomechanical study on flexible intramedullary nail for treatment of pediatric femoral fractures that used similar bone models [27].

When the instrumented models were subjected to mechanical tests using the parameters described above the authors found, as expected, significantly higher relative stiffness in the plate fixation group than in the nail fixation group, both for bending-compression and torsion test.

The results of torsion tests are corroborated by biomechanical studies that compared plates and intramedullary devices. Tencer et al. [31], in a study comparing various methods of stabilization of subtrochanteric fractures of the femur, indicated that intramedullary fixation devices were a maximum of 5% as stiff in torsion as intact bone tested in the same manner, while plate-fixed fractures where nearly 50% as stiff.

Koval et al. [32] in a biomechanical study, which compared the stability of three standard distal femoral fixation techniques, found that an angled plate provided significantly stiffer fixation than antegrade and retrograde locked nails. The authors stressed that, in terms of biomechanics, it is expected the angled plate promote the most stable fixation of a transverse osteotomy because of its high inherent stiffness and the ability to achieve higher compression, minimizing any potential gap at the osteotomy site. Nevertheless, intramedullary nails offer potential biomechanical advantages, namely, less stress on the implant, potential for load sharing, and less tissue dissection. In addition, this kind of implant is more suitable for osteoporotic bone, as seen in the cerebral palsy patient. Also, the periosteum is relatively spared with less disturbance of bone healing.

According to Perren e Claes [33] all fixation methods, except compression techniques, could be seen as flexible fixations. Therefore, they allow some degree of motion between fragments that may improve callus formation or impair bone healing. These authors emphasized that intramedullary nails are located within the mechanical center of the bone and, therefore, have similar mechanical behavior in the frontal and the lateral planes. Considering that nails act as internal splints with load-sharing characteristics, one can assume that the axial stability achieved by the prototype is adequate for secondary bone healing.

The authors who have been adopting techniques of intramedullary fixation for this type of osteotomy in this age group [18,20] emphasize the potential benefits of a minor soft tissue injury and less bleeding, allowing bilateral and concomitant surgery to be better tolerated. The clinical results of these studies showed that the procedures are safe, with fewer problems than those associated with other forms of fixation, and that the results are consistent. Although the implant type and the osteotomy location in the present study are different, the principle of intramedullary fixation as a load-sharing device is the same, as well as the pattern of healing expected.

## Conclusions

The fixation device developed was suitable for insertion into normal and morphologically altered femoral models, and during the simulated rotation osteotomies performed in these models. The stability achieved by the nail, although lower than that achieved by the plate, was found consistent with the type of fixation expected for this class of implant and compatible with the profile of patients with cerebral palsy.

## Competing interests

The authors declare that they have no competing interests.

## Authors' contributions

RGP and JBV designed the nail prototype. RGP implanted the nails and plates, participated in the mechanical testing and drafted the manuscript. RO performed the mechanical testing of the devices. JBV conceived of the study, and participated in its design and coordination and helped to draft the manuscript. All authors read and approved the final manuscript.

## References

[B1] Fabry G MacEwen GD Shands AR Jr Torsion of the femur. A follow-up study in normal and abnormal conditions J Bone Joint Surg Am 1973 55 1726 1738 4804993

[B2] Aktas S Aiona MD Orendurff M Evaluation of rotational gait abnormality in the patients cerebral palsy J Pediatr Orthop 2000 20 217 220 10739285

[B3] Mileski RA Garvin KL Crosby LA Avascular necrosis of the femoral head in an adolescent following intramedullary nailing of the femur. A case report J Bone Joint Surg Am 1994 76 1706 1708 7962031 10.2106/00004623-199411000-00014

[B4] O'Malley DE Mazur JM Cummings RJ Femoral head avascular necrosis associated with intramedullary nailing in an adolescent J Pediatr Orthop 1995 15 21 23 10.1097/01241398-199501000-00005 7883920

[B5] Gonzalez-Herranz P Burgos-Flores J Rapariz JM Lopez-Mondejar JA Ocete JG Amaya S Intramedullary nailing of the femur in children. Effects on its proximal end J Bone Joint Surg Br 1995 77 262 266 7706343

[B6] Raney EM Ogden JA Grogan DP Premature greater trochanteric epiphysiodesis secondary to intramedullary femoral rodding J Pediatr Orthop 1993 13 516 520 10.1097/01241398-199307000-00018 8179649

[B7] Gordon JE Swenning TA Burd TA Szymanski DA Schoenecker PL Proximal femoral radiographic changes after lateral transtrochanteric intramedullary nail placement in children J Bone Joint Surg Am 2003 85-A 1295 1301 12851355 10.2106/00004623-200307000-00016

[B8] Jencikova-Celerin L Phillips JH Werk LN Wiltrout SA Nathanson I Flexible interlocked nailing of pediatric femoral fractures: experience with a new flexible interlocking intramedullary nail compared with other fixation procedures J Pediatr Orthop 2008 28 864 873 10.1097/BPO.0b013e31818e64a1 19034180

[B9] Schall R Estimation in generalized linear models with random effects Biometrika 1991 78 719 727 10.1093/biomet/78.4.719

[B10] Nene AV Evans GA Patrick JH Simultaneous multiple operations for spastic diplegia. Outcome and functional assessment of walking in 18 patients J Bone Joint Surg Br 1993 75 488 494 8496229 10.1302/0301-620X.75B3.8496229

[B11] Norlin R Tkaczuk H One-session surgery for correction of lower extremity deformities in children with cerebral palsy J Pediatr Orthop 1985 5 208 211 3988925

[B12] Saraph V Zwick EB Zwick G Steinwender C Steinwender G Linhart W Multilevel surgery in spastic diplegia: evaluation by physical examination and gait analysis in 25 children J Pediatr Orthop 2002 22 150 157 11856920

[B13] Novacheck TF Gage JR Diplegia and quadriplegia: pathology and treatment The treatment of Gait Problems in Cerebral Palsy 2004 London: Mac Keith Press 345 381

[B14] Kay RM Rethlefsen SA Hale JM Skaggs DL Tolo VT Comparison of proximal and distal rotational femoral osteotomy in children with cerebral palsy J Pediatr Orthop 2003 23 150 154 12604941

[B15] Pirpiris M Trivett A Baker R Rodda J Nattrass GR Graham HK Femoral derotation osteotomy in spastic diplegia. Proximal or distal? J Bone Joint Surg Br 2003 85 265 272 10.1302/0301-620X.85B2.13342 12678365

[B16] Payne LZ DeLuca PA Intertrochanteric versus supracondylar osteotomy for severe femoral anteversion J Pediatr Orthop 1994 14 39 44 10.1097/01241398-199401000-00009 8113370

[B17] Ounpuu S DeLuca P Davis R Romness M Long-term effects of femoral derotation osteotomies: an evaluation using three-dimensional gait analysis J Pediatr Orthop 2002 22 139 145 11856918

[B18] Stevens PM Anderson D Correction of anteversion in skeletally immature patients: percutaneous osteotomy and transtrochanteric intramedullary rod J Pediatr Orthop 2008 28 277 283 10.1097/BPO.0b013e318168d962 18362790

[B19] Zampini J McCarthy JJ Augmented blade plate fixation of a varus derotation osteotomy of the proximal femur using a tension band Orthopedics 2009 32 414 10.3928/01477447-20090511-16 19634820

[B20] Gordon JE Pappademos PC Schoenecker PL Dobbs MB Luhmann SJ Diaphyseal derotational osteotomy with intramedullary fixation for correction of excessive femoral anteversion in children J Pediatr Orthop 2005 25 548 553 10.1097/01.bpo.0000158783.37602.cb 15958913

[B21] Gage JR Cary JM The effects of trochanteric epiphyseodesis on growth of the proximal end of the femur following necrosis of the capital femoral epiphysis J Bone Joint Surg Am 1980 62 785 794 7391102

[B22] Ostrum RF Marcantonio A Marburger R A critical analysis of the eccentric starting point for trochanteric intramedullary femoral nailing J Orthop Trauma 2005 19 681 686 10.1097/01.bot.0000184145.75201.1b 16314714

[B23] Blasier RD Surgical technique for adolescent femur fractures: the trochanteric entry intramedullary nail Oper Tech Orthop 2009 19 24 30 10.1053/j.oto.2009.03.007

[B24] Keeler KA Dart B Luhmann SJ Schoenecker PL Ortman MR Dobbs MB Gordon JE Antegrade intramedullary nailing of pediatric femoral fractures using an interlocking pediatric femoral nail and a lateral trochanteric entry point J Pediatr Orthop 2009 29 345 351 10.1097/BPO.0b013e3181a53b59 19461375

[B25] Filardi V Montanini R Measurement of local strains induced into the femur by trochanteric Gamma nail implants with one or two distal screws Med Eng Phys 2007 29 38 47 10.1016/j.medengphy.2006.01.010 16513407

[B26] Fricka KB Mahar AT Lee SS Newton PO Biomechanical analysis of antegrade and retrograde flexible intramedullary nail fixation of pediatric femoral fractures using a synthetic bone model J Pediatr Orthop 2004 24 167 171 10.1097/01241398-200403000-00006 15076601

[B27] Green JK Werner FW Dhawan R Evans PJ Kelley S Webster DA A biomechanical study on flexible intramedullary nails used to treat pediatric femoral fractures J Orthop Res 2005 23 1315 1320 15961268 10.1016/j.orthres.2005.04.007.1100230612

[B28] Lee SS Mahar AT Newton PO Ender nail fixation of pediatric femur fractures: a biomechanical analysis J Pediatr Orthop 2001 21 442 445 11433153

[B29] Legal H Tonnis D Introduction to the biomechanics of the hip Congenital Dysplasia and Discolocation of the Hip 1987 Berlin: Springer 26 57

[B30] Engel EE Volpon JB Shimano AC Mechanical testing of the tension band wire fixation in the proximal femur Arch Orthop Trauma Surg 1997 116 266 270 10.1007/BF00390050 9177801

[B31] Tencer AF Johnson KD Johnston DW Gill K A biomechanical comparison of various methods of stabilization of subtrochanteric fractures of the femur J Orthop Res 1984 2 297 305 10.1002/jor.1100020312 6491820

[B32] Koval KJ Kummer FJ Bharam S Chen D Halder S Distal femoral fixation: a laboratory comparison of the 95 degrees plate, antegrade and retrograde inserted reamed intramedullary nails J Orthop Trauma 1996 10 378 382 10.1097/00005131-199608000-00003 8854314

[B33] Perren SM Claes L Ruedi TP, Murphy WM Biology and biomechanics in fracture management AO Principles of Fracture Management 2000 New York: Thieme 7 30

